# A mobile app for delirium screening

**DOI:** 10.1093/jamiaopen/ooab027

**Published:** 2021-05-20

**Authors:** Brett Armstrong, Daniel Habtemariam, Erica Husser, Douglas L Leslie, Marie Boltz, Yoojin Jung, Donna M Fick, Sharon K Inouye, Edward R Marcantonio, Long H Ngo

**Affiliations:** University of New England College of Osteopathic Medicine, Biddeford, Maine, USA; The Aging Brain Center, Marcus Institute for Aging Research, Hebrew SeniorLife, Boston, Massachusetts, USA; The Colleges of Nursing and Medicine, The Pennsylvania State University, University Park, Pennsylvania, USA; The Colleges of Nursing and Medicine, The Pennsylvania State University, University Park, Pennsylvania, USA; The Colleges of Nursing and Medicine, The Pennsylvania State University, University Park, Pennsylvania, USA; Division of General Medicine, Department of Medicine, Beth Israel Deaconess Medical Center, Boston, Massachusetts, USA; The Colleges of Nursing and Medicine, The Pennsylvania State University, University Park, Pennsylvania, USA; The Aging Brain Center, Marcus Institute for Aging Research, Hebrew SeniorLife, Boston, Massachusetts, USA; Division of Gerontology, Department of Medicine, Beth Israel Deaconess Medical Center, Boston, Massachusetts, USA; Harvard Medical School, Boston, Massachusetts, USA; Division of General Medicine, Department of Medicine, Beth Israel Deaconess Medical Center, Boston, Massachusetts, USA; Division of Gerontology, Department of Medicine, Beth Israel Deaconess Medical Center, Boston, Massachusetts, USA; Harvard Medical School, Boston, Massachusetts, USA; Division of General Medicine, Department of Medicine, Beth Israel Deaconess Medical Center, Boston, Massachusetts, USA; Harvard Medical School, Boston, Massachusetts, USA; Department of Biostatistics, Harvard T.H. Chan School of Public Health, Boston, Massachusetts, USA

**Keywords:** delirium diagnosis, 2-step delirium protocol, app, XHTML, JavaScript, API, REDCap

## Abstract

**Objective:**

The objective of this study is to describe the algorithm and technical implementation of a mobile app that uses adaptive testing to assess an efficient mobile app for the diagnosis of delirium.

**Materials and Methods:**

The app was used as part of a NIH-funded project to assess the feasibility, effectiveness, administration time, and costs of the 2-step delirium identification protocol when performed by physicians and nurses, and certified nursing assistants (CNA). The cohort included 535 hospitalized patients aged 79.7 (SD = 6.6) years enrolled at 2 different sites. Each patient was assessed on 2 consecutive days by the research associate who performed the reference delirium assessment. Thereafter, physicians, nurses, and CNAs performed adaptive delirium assessments using the app. Qualitative data to assess the experience of administering the 2-step protocol, and the app usability were also collected and analyzed from 50 physicians, 189 nurses, and 83 CNAs. We used extensible hypertext markup language (XHTML) and JavaScript to develop the app for the iOS–based iPad. The App was linked to Research Electronic Data Capture (REDCap), a relational database system, via a REDCap application programming interface (API) that sent and received data from/to the app. The data from REDCap were sent to the Statistical Analysis System for statistical analysis.

**Results:**

The app graphical interface was successfully implemented by XHTML and JavaScript. The API facilitated the instant updating and retrieval of delirium status data between REDCap and the app. Clinicians performed 881 delirium assessments using the app for 535 patients. The transmission of data between the app and the REDCap system showed no errors. Qualitative data indicated that the users were enthusiastic about using the app with no negative comments, 82% positive comments, and 18% suggestions of improvement. Delirium administration time for the 2-step protocol showed similar total time between nurses and physicians (103.9 vs 106.5 seconds). Weekly enrollment reports of the app data were generated for study tracking purposes, and the data are being used for statistical analyses for publications.

**Discussion:**

The app developed using iOS could be easily converted to other operating systems such as Android and could be linked to other relational databases beside REDCap, such as electronic health records to facilitate better data retrieval and updating of patient’s delirium status.

**Conclusion:**

Our app operationalizes an adaptive 2-step delirium screening protocol. Its algorithm and cross-plat formed code of XHTML and JavaScript can be easily exported to other operating systems and hardware platforms, thus enabling wider use of the efficient delirium screening protocol that we have developed. The app is currently implemented as a research tool, but with adaptation could be implemented in the clinical setting to facilitate widespread delirium screening in hospitalized older adults.

LAY SUMMARY

Delirium among hospitalized patients is an important clinical problem. Up to one-third of hospitalized older adults experience delirium, yet over half of cases go unrecognized. Patients who develop delirium have an increased risk of death, institutionalization, and cognitive decline. The estimated annual costs of delirium in the United States are $164 billion. Due to delirium’s adverse effects on patients and the healthcare system, it is critical to have efficient and accurate methods for screening and identifying delirium. We developed an iPad app that facilitated administration of an adaptive two-step protocol for delirium diagnosis and assessed its usability among three types of clinicians (doctors, nurses, and certified nurse assistants) at two hospitals. In this article, we describe technical details of the iOS-based app. Our app can be easily adapted to other operating systems such as Android, Microsoft Windows, or Linux. The provided technical details of the code can be modified for new apps suitable for other clinical applications.

## INTRODUCTION

### Background and significance

Delirium among hospitalized patients is an important clinical problem. Up to one-third of hospitalized older adults experience delirium, yet over half of these cases are unrecognized by the treating clinicians.^1^ In the hospital, delirium has been linked to complication, and prolonged length of stay. For long-term outcomes, delirium is known to be associated with an increase in mortality, dementia, acute cognitive decline, and institutionalization. Delirium costs the healthcare system over $164 billion in the United States annually, with European nations collectively paying $182 billion in associated costs yearly.^2–4^ Due to delirium’s adverse effects on patients and the healthcare system, there is a need for efficient and accurate methods for screening and identifying delirium.^5^

Although delirium is recognized as a serious clinical problem, it remains under-recognized and underdiagnosed. One study found that 61% of delirium diagnoses were missed by the patients’ primary hospital teams, while other reviews found similar percentages of missed diagnosis.^6^^,^^7^ Various factors attribute to the prevalence of missed delirium diagnoses, including limited understanding of delirium, insufficient application of delirium assessments, and the need for substantial training on the assessments.^8^^,^^9^ To address these concerns, Marcantonio et al^9^ previously developed the 3-Minute Diagnostic Assessment for Confusion Assessment Method (3D-CAM) to diagnose delirium.

The 3D-CAM operationalizes the CAM diagnostic algorithm, which is the most commonly used tool for diagnosis of delirium both in clinical and research settings.^10^ The CAM algorithm considers 4 features of delirium: (1) acute change or fluctuating course, (2) inattention, (3) disorganized thinking, and (4) altered level of consciousness. The diagnosis of delirium requires the presence of features 1, 2, and either 3 or 4. In the 3D-CAM, each CAM feature is assessed using cognitive testing, patient symptom report, and interviewer observational items. The presence of just 1 incorrect answer, patient symptom, or positive interviewer observation is sufficient to trigger the presence of the CAM feature. Marcantonio et al^9^ prospectively validated the 3D-CAM in a cohort of 201 older general medicine patients. Compared with an independent clinical reference standard, the 3D-CAM was highly accurate, with 95% sensitivity, and 94% specificity.^9^ The strengths of the 3D-CAM are (1) its rigorous derivation using Item Response Theory, (2) its prospective validation in 201 older adults using a clinical reference standard,^9^ and (3) its excellent test characteristics, with 95% sensitivity and 94% specificity. Weaknesses are (1) validation was conducted at a single site; (2) patients were assessed only on a single day, during the day shift; and (3) only a paper and pencil version of the instrument was used, a limitation that is addressed in the current study.

While the 3D-CAM substantially shortened and simplified assessment of delirium, clinicians requested still shorter approaches for widespread screening. To address this need, Fick et al^5^ identified 2 questions from the 3D-CAM, “What is the day of the week?” and “What are the months of the year backwards?” which when paired together were found to have high sensitivity for detecting delirium. This ultrabrief screener, termed the UB-2, can be completed in 30–40 seconds and can be used to quickly rule out the presence of delirium in patients who are able to answer both questions correctly. The UB-2 only has modest specificity, and “positive” screens require further assessment.^5^^,^^11^

Fick et al^12^ have subsequently proposed a 2-step approach for delirium identification, in which the UB-2 is administered first, followed by the 3D-CAM in those who get 1 or both questions incorrect. To further shorten the assessment, we have introduced a skip pattern for the 3D-CAM. Since a single incorrect answer to a cognitive test, or positive report of a delirium symptom, triggers presence of a CAM feature, all subsequent questions mapped to that feature can be skipped. The 2-step protocol of the UB-2 plus 3D-CAM with skip is an example of computer adaptive testing, in which answers to previous questions determine the next series of questions to be administered.

Mobile apps have been increasingly utilized in healthcare, with many focused on patient education, health behaviors, and disease management.^13^^,^^14^ There are few apps specifically built for delirium detection and management. Among those currently available, the apps are aimed at clinician education on delirium and its management.^15–17^ Two additional apps operationalize the CAM-ICU assessment, the most widely used CAM identification tool in intensive care unit settings.^18^^,^^19^ However, there are currently no apps for the UB-2 or 3D-CAM, nor one that combines the 2 as described above. To facilitate implementation of this 2-step, adaptive delirium identification protocol, we aimed to develop a portable, user-friendly, and efficient mobile application (app) embedding both the UB-2 and 3D-CAM, as part of the Researching Efficient approaches to Delirium Identification (READI) project.^5^^,^^12^ In addition to facilitating administration of the 2 tools described above, we aimed to have our app connect directly to the secure web–based Research Electronic Data Capture (REDCap) database (described in “Methods” section) using the REDCap application programming interface (API). We are not aware of any other delirium-focused clinical app using this interface. For the data collection vehicle, we decided on using the iPad Air tablet. Previous studies have utilized iPad tablets for data collection, and clinicians are more frequently using iPad tablets in their clinical practice.^20–22^ We found that the iPad Air tablet provided the optimal combination of portability, user-friendliness, and reliability.

### Objectives

In this article, we had the following aims: (1) describe the data collection and management system for the 2-step algorithm for delirium diagnosis, (2) describe the technical implementation of the app with detailed explanation of the extensible hypertext markup language (XHTML), and JavaScript code, and (3) report qualitative data assessing usability of the app and illustrate the use of data collected from the app for the analysis of delirium administration time. The ultimate goal of the app is to provide a user-friendly interface for an adaptive assessment for delirium that reduces assessment time while maintaining diagnostic accuracy. In this article, we described the app’s technical aspects.

## MATERIALS AND METHODS

### Participants

The cohort includes 535 hospitalized patients with mean age 79.7 years admitted to the medicine service at a large urban academic medical center and a smaller rural community hospital. The study field staff were given permission to screen hospitalized patients aged 70 and older and approach eligible patients for informed consent to participate in the study. The IRB of all participating institutions granted approval for this study. The mean age of the patient cohort was 79.7 years (SD = 6.6). Forty-three percent of participants are male, and 88% are white. Fifty-three percent of the patients have some college or higher education. Forty-three percent are married.

### Delirium screening procedure

After obtaining patient (or proxy) informed consent and enrollment in the study, a trained researcher performed a reference standard delirium assessment for delirium. The reference standard delirium assessment conducted by the senior researcher is a full structured delirium assessment which includes assessment of cognitive function using the Mini-Mental State Examination (purchased from Psychological Assessment Resources), supplemented with Digit Span for additional attention testing, along with the Delirium Symptom Interview (DSI) to elicit patient symptoms of delirium. Using data from the cognitive testing and DSI, the senior researcher will complete the long Confusion Assessment Method (CAM), which includes a diagnosis of delirium using the CAM diagnostic algorithm. This detailed assessment, which takes 20–30 minutes served as the reference standard. Thereafter, the patient’s physician, nurse, and certified nursing assistant (CNA) performed the app-facilitated, adaptive 2-step delirium identification protocol described above.^5^^,^^12^ Clinicians were randomized each day to either the UB-2 plus full 3D-CAM or 3D-CAM with skip pattern. This is further described in a later section, “REDCap Database Set-up.” Each study site recruited as many as 300 clinical staff (physicians, nurses, and certified nurse assistants) who used the app to administer the delirium identification protocol. Participating clinicians of physicians, nurses, and CNAs were consented. In addition to our patients, these clinicians were also treated as study subjects and gave their full consent to participate in this study.

### READI data collection system

**Figure 1.**
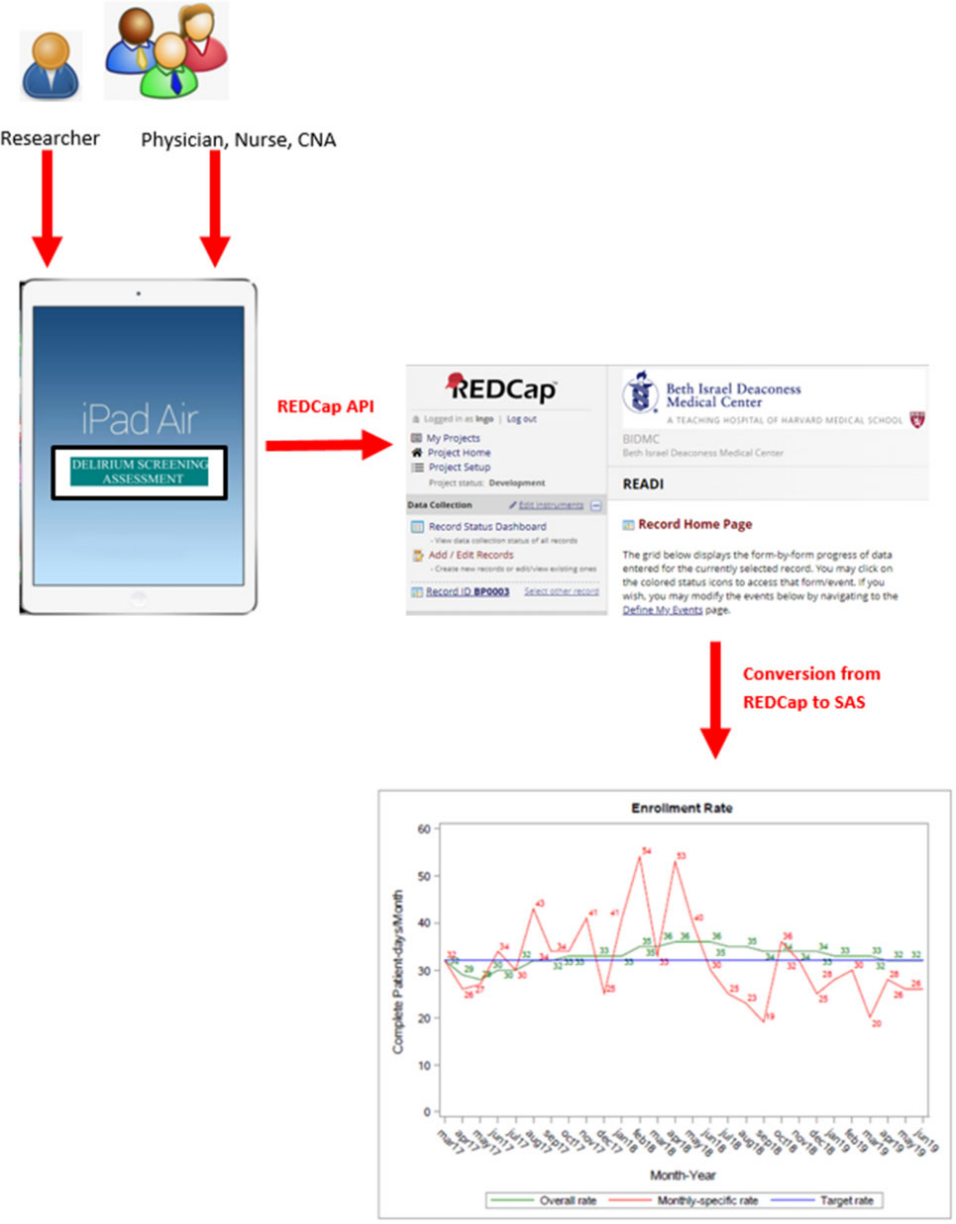
Data flow of the READI Project: from Staff to iPad to REDCap and to SAS for analysis.

Figure 1 shows the data flow of the READI study. The data entered by the clinician app users are first entered into the iPad Air where the app resides. The entered data are captured and stored in the random access memory (RAM) of the machine (which holds a 64 Gigabyte flash drive). When the assessment is complete, an upload action triggered by the user sends the locally stored variables from the app to the corresponding data fields in REDCap relational database system. The mapping of the data between the iPad RAM and REDCap database is made possible using the REDCap API. The third component of the data collection system is the Statistical Analysis System (SAS), which processes the data from the REDCap database for reporting and analysis. The REDCap system has a download function that allows data to be converted to the SAS data format. The SAS data are used weekly to assess the study enrollment status, and other important clinical parameters that the team would like to track during the data collection, for example, the number of patients enrolled to date, and the prevalence of delirium and dementia. In the following sections, we further discuss the app, its implementation, and assessment of its usability and application.

### App development

*Selection of mobile device:* prior to app development, the team reviewed options for portable devices. We focused on the Apple iPad and Microsoft Surface tablet. The team emphasized choosing the tablet that would optimize portability and screen size. We chose the Apple iPad Air as we felt that this tablet provided the best combination of portability, screen size, speed, and reliability. Furthermore, the iPad was already in use in clinical operations at the study hospitals, and thus was familiar to many study clinicians. The app was written in XHTML and JavaScript, which is compatible and portable to other operating systems such as Microsoft Windows 10.

*Selection of relational database system for study data:* data collection for the READI project occurred in REDCap, a secure, web–based application that helps build and run online surveys and databases.^23^ Various features of REDCap include an interface for validated data entry, audit trails for documenting data changes and exports, exporting procedures for downloading research data to numerous statistical packages including SAS, and securely importing data from external sources.^23^ REDCap also has an app version for use on portable devices. Initially, we attempted to build the delirium identification protocol using the web–based app interface provided by REDCap. However, the team found that there were limitations in the font, color, and layout of the survey. Additionally, the REDCap app design specification was not compatible for our assessment’s design, including the novel skip pattern algorithm described above, and could not provide direct linkage of the app to the REDCap database. The team felt these limitations affected the user-friendliness, ease, and efficiency of the screening tool. We ultimately decided to create a separate app that would use a more flexible, common platform tool such as XHTML and JavaScript to link with REDCap.

Therefore, the READI app was created using the REDCap API. Coding was performed by one of the authors (D.H.) using XHTML and JavaScript, and we collaborated with our institutional REDCap administrator to receive authorized access via the REDCap API Token to link our iOS–based iPad READI app to the REDCap system.

**Figure 2.**
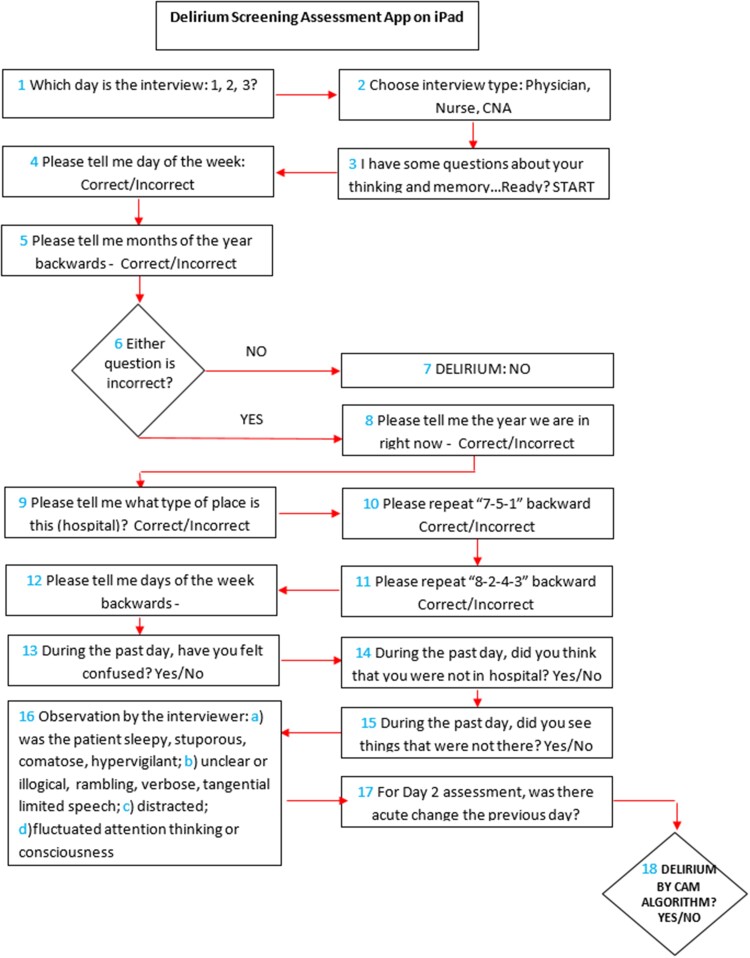
Flow diagram of the content of the delirium screening assessment app.

*Algorithm of delirium screening for the app*: the coding of the READI app followed the algorithm described in Figure 2, which contains 18 key steps. Upon clicking the READI app icon on the iPad, the authorized user is asked on which day the interview is occurring (box 1) (most patients are interviewed on 2 consecutive days after hospital admission (days 1 and 2), although a few are also interviewed on day 3). The next question is whether the interview is being performed by a physician, nurse, or CNA (box 2). Box 3 prompts a question to get the patient ready for the screening questions (UB-2), which are asked in boxes 4 and 5 (feature 3). If both questions are answered correctly (box 6) then the patient is screen negative for delirium and does not need to answer any more questions (box 7). If the patient gets 1 or both of the UB-2 screen questions wrong, there are 2 possible scenarios: (1) the full 3D-CAM, which means answering additional patient questions from boxes 8 to 17 or (2) 3D-CAM with skip pattern, which is a subset of questions and observation items from boxes 8 to 17. As described above, the skip pattern allows questions within each of the 4 CAM diagnostic features to be skipped if one of the questions is answered incorrectly. Both scenarios allow determination of the presence or absence of each of the 4 features in the CAM algorithm (box 18) to determine the presence or absence of delirium. An additional feature of the app is implemented on the day 2 or 3 assessments (box 17). The app automatically compares the current day’s answers with the stored REDCAP variables from the previous day. Any new incorrect answer to a cognitive question or a new “positive” patient symptom or interviewer observation triggers CAM Feature 1, Acute Change.

We collected qualitative data on the usability of the app on a subset of clinicians (physicians, nurses, and certified nurse assistants), recently published in Ref..^24^ Briefly, 50 physician hospitalists, 189 nurses, 83 certified nurse assistants participated in a 14-month period during which trained field researchers collected data (767 observations) on patient environment, staffing level, administration of the 2-step protocol, and experiences of the clinical staff during the protocol implementation. Immediately after the app–based assessments, the field team staff also conducted 231 brief semistructured interviews of clinical staff to obtain their views about the protocol, patient experience, the app, and other potential barriers and facilitators. Interviews were conducted face-to-face, were recorded and stored and ranged in duration from 5 to 10 minutes. Analyses were conducted in parallel with ongoing observations and interviews to evaluate for thematic saturation. A subset of provider quotes that represent direct feedback about the app are presented. The comments were categorized into positive comments versus suggestions or nonpositive comments.

Quantitative data included assessment of delirium administration time. To determine the time required to complete the assessments, the app integrated a timing feature that recorded a time stamp each time an assessment answer was entered into the iPad. It also included a “pause/resume” button in case the assessment was interrupted, such as by the arrival of another clinician or family member. We used the system automated time stamp data (start time, and stop time) to compute the duration for each question and answer and for the entire session of the assessment. Delirium administration time analysis was based on these time durations. We compared the distributions of these times among the 3 types of clinicians and for protocols consisting of the UB-2 only, and the 2-step protocols (UB-2 and 3D-CAM).

## RESULTS

There are 3 types of results are (1) the graphical interface of the apps and the associated XHTML and JavaScript code, (2) qualitative data on app usability during clinician interviews, and (3) quantitative data on delirium administration.

### Graphical interface

We extracted important parts of the XHTML and JavaScript code for the app and present them in Supplementary Figures S3–S9, along with a picture of the corresponding graphical display for the app. Supplementary Figure S1 shows the invoking of the XHTML and JavaScript code from our server and the customization of the initial screen of the READI app. The code allows us to have full interface design flexibility in terms of font size, color, placement of objects, and logical coding, all of which would be more difficult if we used the more restricted REDCap design tools. In Supplementary Figure S2, we present a typical screen for the app, which shows a question for screening, “Please tell me the day of the week.” (box 4 of Figure 2). The user’s response is coded as correct or incorrect and is stored in the iPad’s RAM using the XHTML variable VAR2, highlighted in the code. These data are formatted according to the REDCap’s API requirement before being uploaded into the READI REDCap database. In Supplementary Figure S3, we show how the app is linked to the REDCap database via the use of a REDCap API. The value of the token, highlighted, was given by the REDCap administrator specifically for the app to have read and write capability from and to the REDCap database. While the primary purpose of the app is to write into RedCAP, as described above there is a specific function related to delirium testing that requires reading of previous 3D-CAM results—to assess for acute change (CAM feature 1). Here in this figure, the app retrieves the variables from the REDCap database (b1, b2, to b21). These variables store the responses of the 3D-CAM questions mentioned in Figure 2 in REDCap from the previous assessment. The app, via the API token, compares previous REDCap variable values to the current app variables (var1, var2, and var21) using the JavaScript conditional statement IF-THEN-ELSE to determine if the patient has any new “incorrect” or “positive” responses, indicative of an acute change. The use of conditional statements is also employed in the code for the 3D-CAM skip pattern. Supplementary Figure S4 demonstrates the code that we use to format data from the app prior to uploading into REDCap. The statement *this.form==1* refers to data collected from day 1 by the CNA. The code following the statement *if this.form==1* shows how the CNA’s screening data for the 2 questions (var2 and var7) and their time stamps (time2 and time7) are formatted to be uploaded to REDCap. Similarly, data from the physician (*this.form==2*) with the app variables var1 to var22 are mapped to REDCap variables b1 to b22. The time stamps time0 to time22 from the app (time at which the variable var1 to var22 entered responses) are also mapped to the REDCap time variables bt0 to bt22. For the nurse’s data, app variables var1 to var22 are mapped to REDCap variable n1 to n22, and the corresponding time stamps from the app nt0 to nt22 mapping to REDCap variable time0 to time22. Supplementary Figure S5 shows as an example using the CNA data and how the app data, once formatted, can be sent to REDCap. Here the CNA data from days 1 and 2 are sent to the REDCap database, and the statement “var request=$.post(apiUrl, token: token, content: record, format: “xml” type: “flat”, data: xml)” sends the request for upload to the REDCap database. This part of the code is used often to send app data to REDCap. Supplementary Figure S6 presents the important code determining the final delirium status via the JavaScript *myFunction()*. This function is called when the delirium assessment from the physician or nurse is finished for days 1, 2, 3 or for either the 3D-CAM or skip-pattern 3D-CAM scenario. The code executes the CAM algorithm (the last IF-THEN-ELSE statement) using the data from the 4 features and renders delirium status either POSITIVE or NEGATIVE at the end on the last app screen when the “Upload Patient Data” button is clicked (Supplementary Figure S7).

### Qualitative data to assess the app usability

Based on the semistructured interviews of clinical staff immediately after completing the app–based protocol, the majority of clinicians thought favorably of the app, and found it very useful and easy to use. They offered comments such as “app is great, especially liked the big letters and the big buttons,” “liked the dialog box,” “really liked the skip pattern,” and “app is very useful,” and “super easy.” The issues raised by several users are minor and can be easily addressed. For example, reordering of the skip pattern 3D-CAM should be considered, and the 2 screening questions may not be effective on day 2 since the patient could remember the answers from day 1. But these comments relate more to the content and structure of the 2-step delirium identification protocol and not to the READI app itself. Additional qualitative data are available in our recent publication.^24^

**Table 1.** Weekly generated report using the app data on enrollment of patients^a^

	Site 1	Site 2	Both sites
Number of enrolled patients	270	265	535
Number of patients who have day 1 research assessment completed	269	257	526
Number of patients who have day 2 research assessment completed	186	205	391
Number of patients who have day 3 research assessment completed	2	16	18
Number of patients who have only 1 day research assessment completed	82	48	130
Number of patients who have 2 days research assessment completed	187	210	397
Delirium (%)	46 (17)	71 (27)	117 (22)
Dementia (%)	80 (30)	107 (40)	187 (35)
Number of reference standard assessments from enrolled patients	456	468	924
Number of patients who have day 1 assessment completed	256	241	497
Number of patients who have day 2 assessment completed	178	189	367
Number of patients who have day 3 assessment completed	2	15	17
Number of patients who have only 1 day of assessment completed	90	71	161
Number of patients who have 2 days of assessment completed	173	187	360
Total number of assessments completed	436	445	881
Number of patients with complete proxy interview	181	251	432

Each patient has 2 delirium assessments done on 2 different days. However, not all patients have 2 assessments. Patients who missed day 2 assessment may also have day 3 assessment done.

### Quantitative data to track study progress and to assess delirium administration time

We collected quantitative data about the patient’s delirium assessment (Table 1), and the duration of time (in seconds) each question and answer takes. Our clinicians performed a total of 881 assessments on 535 patients. We tracked the number of assessments through time, and the tracking data were examined weekly because we generated detailed reports of enrollment tracking, demographics, and outcome prevalence (eg, delirium and dementia rate). We have examined the efficiency issue by analyzing the delirium administration time among the 3 types of clinicians. Table 2 shows the results for the delirium administration time. All results are significantly shorter than the 3D-CAM, which was over 3 minutes.

**Table 2.** Distribution of delirium administration time by clinician type

Type	Clinician type	*N*	Mean (SD) (s)	Median (Q1, Q3) (s)
UB-2	CNA	862	62.0 (51.3)	49 (35, 74)
	Nurse	873	55.2 (33.5)	46 (32, 70)
	Physician	856	55.2 (43.9)	44 (31, 66)
2-Step	Nurse	866	103.9 (99.3)	60 (34, 143.3)
	Physician	854	106.5 (105.4)	62 (33, 155)

*Note*: UB-2: ultrabrief with 2 questions asked, and if the patient answers incorrectly 1 or 2 questions, then the patient goes to the next step assessed by either the full 3D-CAM or 3D-CAM with skip pattern. 2-Step: UB-2 plus 3D-CAM. *N*: number of total assessments with most patients had 2 assessments. Q1, Q3: 25th percentile and 75th percentile.

*Abbreviation:* SD: Standard deviation.

## DISCUSSION

With the goal of improving delirium detection on the hospital wards, we set out to develop and implement a user-friendly, efficient READI app on the iOS–based iPad platform that operationalized a computer adaptive 2-step delirium identification protocol. Considering our team’s focus on optimizing speed and responsiveness for the app and its connection with REDCap, we chose to code the app using XHTML and JavaScript. When deciding between a REDCap plug-in versus a stand-alone app, our team chose the stand-alone app to optimize the user experience. The READI app is externally hosted with an API that communicates with the REDCap database, using XHTML for the user interface, and uses hypertext preprocessor API for linking the app to the REDCap database. Rather than using REDCap’s data entry trigger, our app is based on runtime and is event-triggered; thus, the user triggers data entry. There are also passive timing measures embedded in the app, which are utilized in the analytic phase of the project. Based on our experience with over 2700 assessments performed on over 500 hospitalized older adults, the stand-alone app provides speed and responsiveness, is user-friendly, and has captured data with excellent quality.

Our qualitative assessment of the usability of the app demonstrates that users were highly satisfied. A minority of user’s comments related to suggestions on how to improve the app. Continuous feedback from the clinical and field team also helped to improve the interface design and the addition of clinically relevant variables to the app. We use standard tools of XHTML and JavaScript and thus our app is highly portable to different operating systems such as Android, Linux, and other types of portable devices such as smart phones.

Quantitative data collected by the app allowed us to assess delirium administration time and compare this time across types of clinicians and between different protocols such as 2-step with full 3D-CAM versus 3D-CAM with skip pattern.

There are few published articles on app development for delirium research. Of the studies that we mentioned above^14–19^ that are relevant to delirium research, none was about delirium screening, and none offers technical details of the app development process, or the associated programming code. To our knowledge, our article is the first to address this gap in the literature.

With modifications, we believe that the READI app can be used to enter data into other relational electronic health record (EHR) databases. This operability will be important to facilitate widespread adoption of this app into clinical practice. We think that for any EHR–based relational database system, the key is in the implementation of the API similar to the one we described in detail in our code for linking the app with REDCap. This linkage allows the flow of data back and forth between the app and the EHR system*.* While we believe our work has substantially advanced the field, it still represents an early effort, and further improvements are possible. We decided to include important key pieces of the app’s code in the hope that others could build on our interface design and logic for future app development and improvement. Feedback on the technical details of the code would be welcomed so that we can improve our experience in developing similar work for future studies.

We note that the planned use of the app is for systematic screening of all hospitalized older adults, not for detailed diagnostic evaluations. The app’s innovation are its user friendly interface that allow users (clinicians) to quickly screen out patients who do not need to be given full delirium assessment, and its technical implementation was based on universal tools of XHTML, and JavaScript that allow to app to be easily converted to different hardware platforms, and with the use of a standard API, the app can be linked to different database system for integration with other sources of patient’s data.

Our work is related to computer adaptive testing which is still underdeveloped for delirium. The benefit of computer adaptive testing is shorter administration time and therefore less cost. The drawback is less information about the patient’s overall state of delirium, and measures such as delirium severity cannot be reliably estimated. Also although we designed the READI app for a research project, the patients and clinicians were consented, the app-guided assessments were performed by real clinicians, not by researchers. Therefore, the results are more informative toward clinical practice than a study conducted solely by researchers. This is the translational value of the app.

Based on the experiences and the feedback from the clinicians who participated in the study, we recognize several limitations of this app. First, although the interface of the app is acceptable to users, it is somewhat cluttered at the top due to iOS system features. The advancement of the screens could be better scrolled instead of paged. So in general, for interface designed could be improved. We are already in the process of redesigning the interface using native iOS Application Developer tools instead relying on XHTML and JavaScript. The app is currently linked to our institution research–based RedCAP database, but is not linked to the EHR. The automated linkage between the app and EHR would be more efficient for data retrieval and direct updating of the patient’s data in the app. We have recognized these issues and are working on improvement in the next version of the app. In this project, our primary aim was research focused on evaluating the feasibility, efficiency, and acceptance of the app. Thus, the lack of design specifications for wider integration of the app into institutional EHRs is a limitation. However, that remains our ultimate goal. We are currently working with information technology experts at our institutions to scale up the app.

## CONCLUSION

This manuscript presents our development and early experience with an iPad–based app to facilitate administration of a 2-step delirium identification protocol that employs computer adaptive testing to maximize efficiency. XHTML and JavaScript are excellent standard tools for the development of data entry apps that can be linked to a relational database such as REDCap. Our app for efficient delirium screening shows that by using an API, a fully flexible, functional, and user-friendly app can be developed and linked to a REDCap database to facilitate real time data capture. Our app can be adapted for use in other operating systems and hardware platforms, thus enabling systematic bedside screening of delirium in vulnerable hospitalized older adults.

## FUNDING

This work was funded by the following grants: R01AG030618 (DMF and ERM), K24AG035075 (ERM), R24AG054259 (SKI), and P01AG031720 (SKI) all from the National Institute on Aging.

## AUTHOR CONTRIBUTIONS

ERM and DMF contributed to conception or design of the work. BA, ER, MB, DMF, and ERM contributed to data collection. LHN, DH, YJ, ERM, MB, DMF, and DLL contributed to data analysis and interpretation. BA, DH, ERM, SKI, and LHN contributed to drafting the article. All authors contributed to critical revision of the article and final approval of the version to be published.

## SUPPLEMENTARY MATERIAL

Supplementary material is available at *Journal of the American Medical Informatics Association* online.

## Supplementary Material

ooab027_Supplementary_Data

## Data Availability

The data underlying this article will be shared on reasonable request to the corresponding author.
