# Supplementary figures and images for: A mobile app for delirium screening
Source: JAMIA Open. 2021 May 20;4(2):ooab027. doi: 10.1093/jamiaopen/ooab027 (PMC8446432; doi:10.1093/jamiaopen/ooab027)

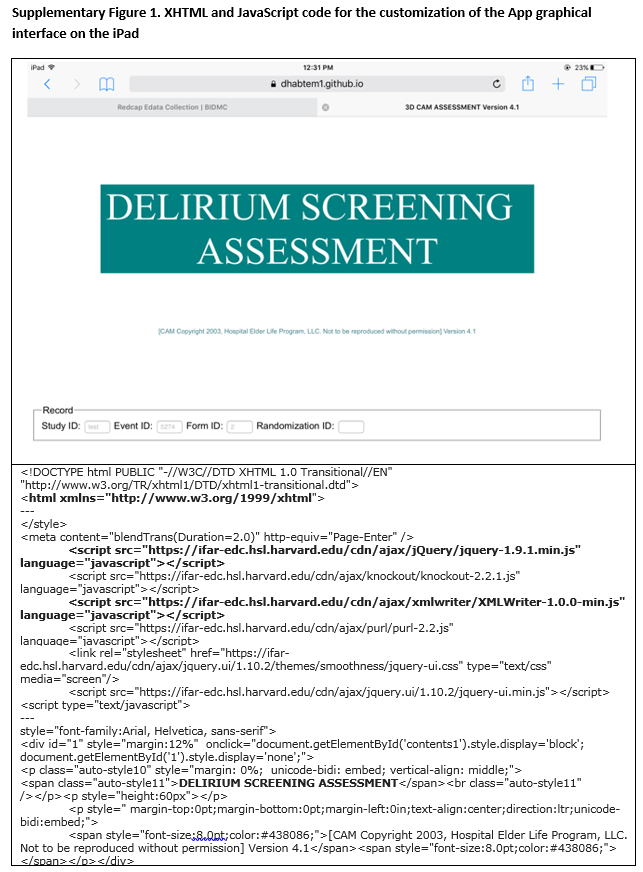

Supplement: ooab027_Supplementary_Data [file ooab027_supplementary_data.zip › supplementary figure 1.png]

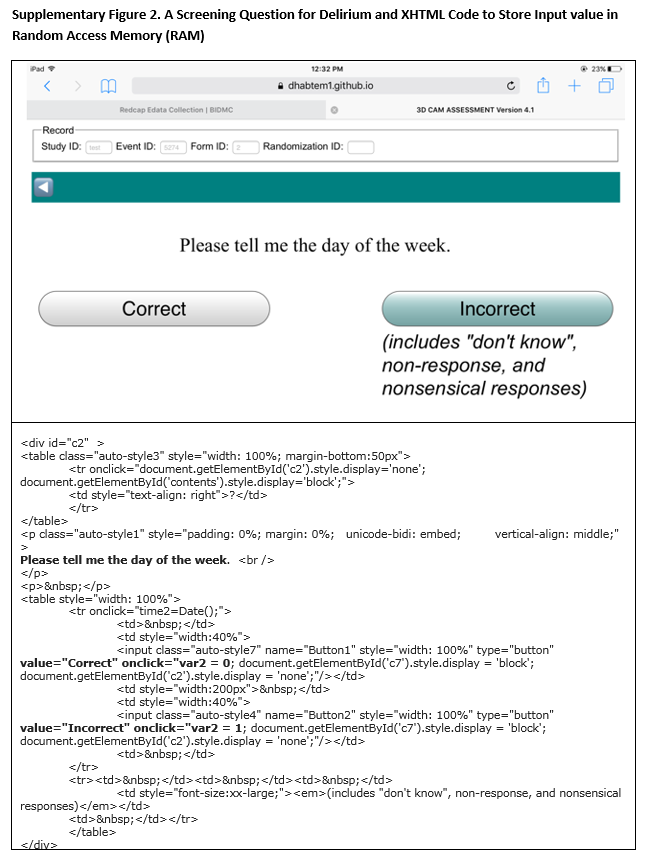

Supplement: ooab027_Supplementary_Data [file ooab027_supplementary_data.zip › supplementary figure 2.png]

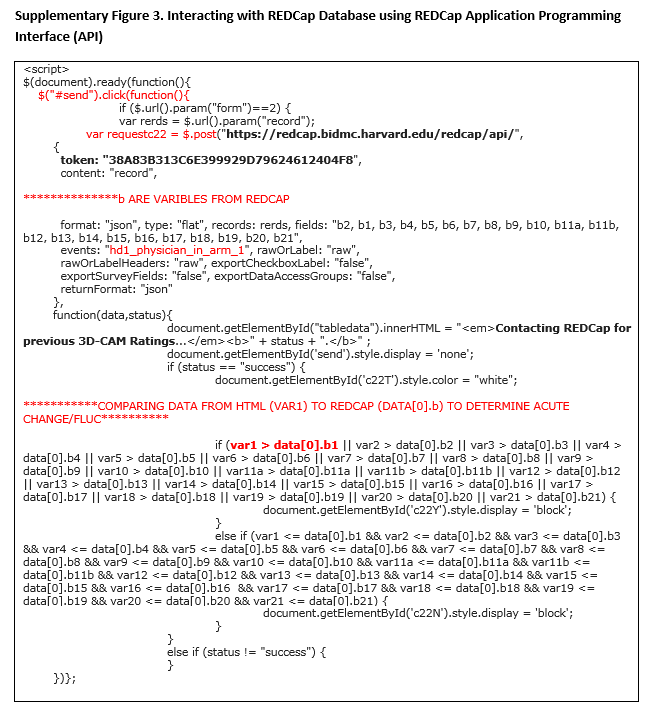

Supplement: ooab027_Supplementary_Data [file ooab027_supplementary_data.zip › supplementary figure 3.png]

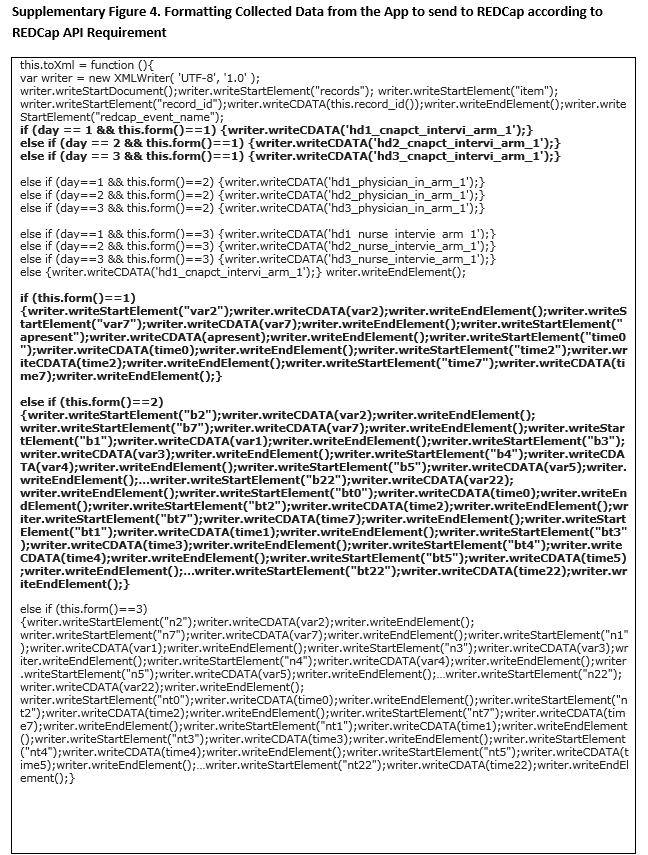

Supplement: ooab027_Supplementary_Data [file ooab027_supplementary_data.zip › supplementary figure 4.png]

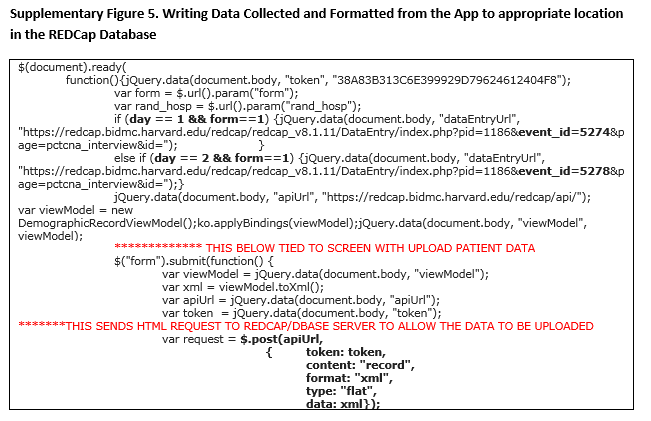

Supplement: ooab027_Supplementary_Data [file ooab027_supplementary_data.zip › supplementary figure 5.png]

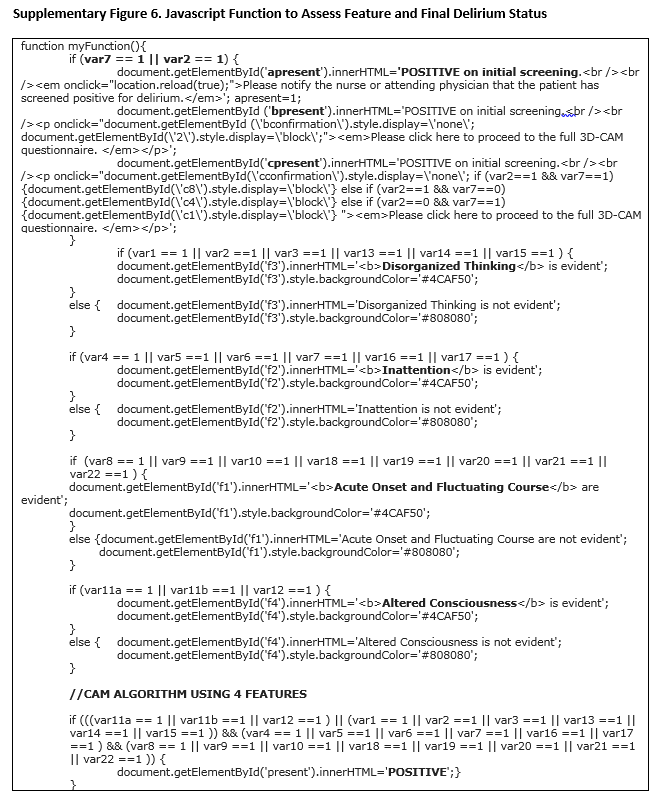

Supplement: ooab027_Supplementary_Data [file ooab027_supplementary_data.zip › supplementary figure 6.png]

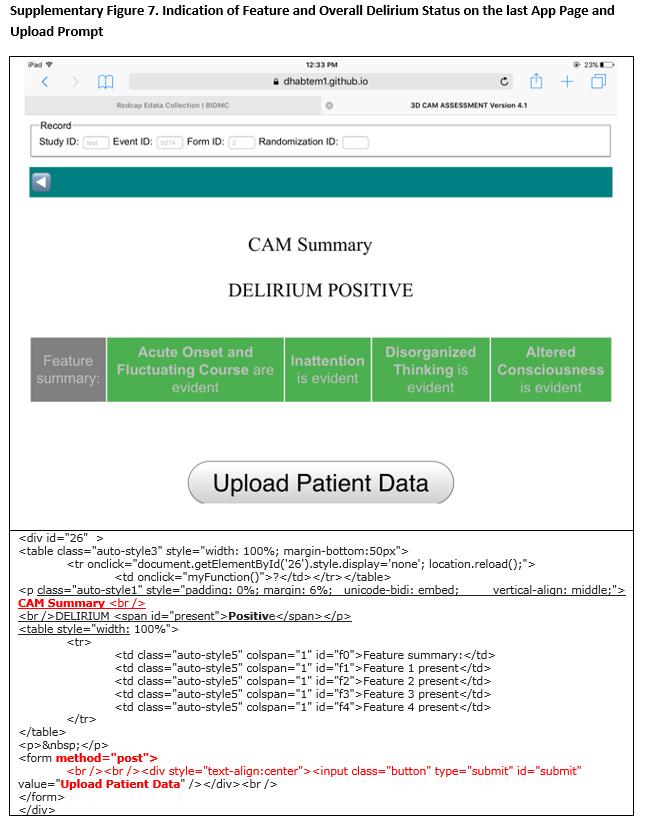

Supplement: ooab027_Supplementary_Data [file ooab027_supplementary_data.zip › supplementary figure 7.png]
